# Supplementary material for: A modular transcriptional signature identifies phenotypic heterogeneity of human tuberculosis infection
Source: Nat Commun. 2018 Jun 19;9:2308. doi: 10.1038/s41467-018-04579-w (PMC6008327; doi:10.1038/s41467-018-04579-w)
Supplement: Supplementary file 1 — Supplementary Information [file 41467_2018_4579_MOESM1_ESM.pdf]

Supplementary data

A modular transcriptional signature identifies phenotypic heterogeneity of human tuberculosis infection

Akul Singhania[1,#], Raman Verma[2,#], Christine M. Graham[1], Jo Lee[2], Tran Trang[3], Matthew Richardson[2], Patrick Lecine[3], Philippe Leissner[3], Matthew P.R. Berry[4], Robert J. Wilkinson[5,6,7], Karine Kaiser[8], Marc Rodrigue[8], Gerrit Woltmann[2], Pranabashis Haldar[2,¶], Anne O'Garra[1,9,¶]

[1] Laboratory of Immunoregulation and Infection, The Francis Crick Institute, London, United Kingdom.
[2] Respiratory Biomedical Research Centre, Institute for Lung Health, Department of Infection Immunity and Inflammation, University of Leicester, Leicester, United Kingdom.
[3] BIOASTER Microbiology Technology Institute, Lyon, France.
[4] Department of Respiratory Medicine, Imperial College Healthcare NHS Trust, St Mary's Hospital, London, United Kingdom.
[5] Wellcome Centre for Infectious Diseases Research, Africa; Institute for Infectious Diseases and Molecular Medicine, University of Cape Town, Observatory 7925, South Africa.
[6] Department of Medicine, Imperial College London, London, United Kingdom.
[7] Tuberculosis Laboratory, The Francis Crick Institute, London, United Kingdom.
[8] Medical Diagnostic Discovery Department, bioMérieux SA, Marcy l'Etoile, France.
[9] National Heart and Lung Institute, Imperial College London, London, United Kingdom.

[#]contributed equally to this work.
[¶]co-supervised the work equally.

Correspondence and requests for materials should be addressed to AOG (email: Anne.OGarra@crick.ac.uk)

Supplementary Figures

Supplementary Figure 1. Schematic diagram documenting the objectives undertaken in the study. The cohorts analysed as part of each objective are shown, with microarray and RNA-seq cohorts represented in different colours. Table numbers (for the patient numbers/clinical characteristics for each dataset), and figure numbers associated with each objective are shown.

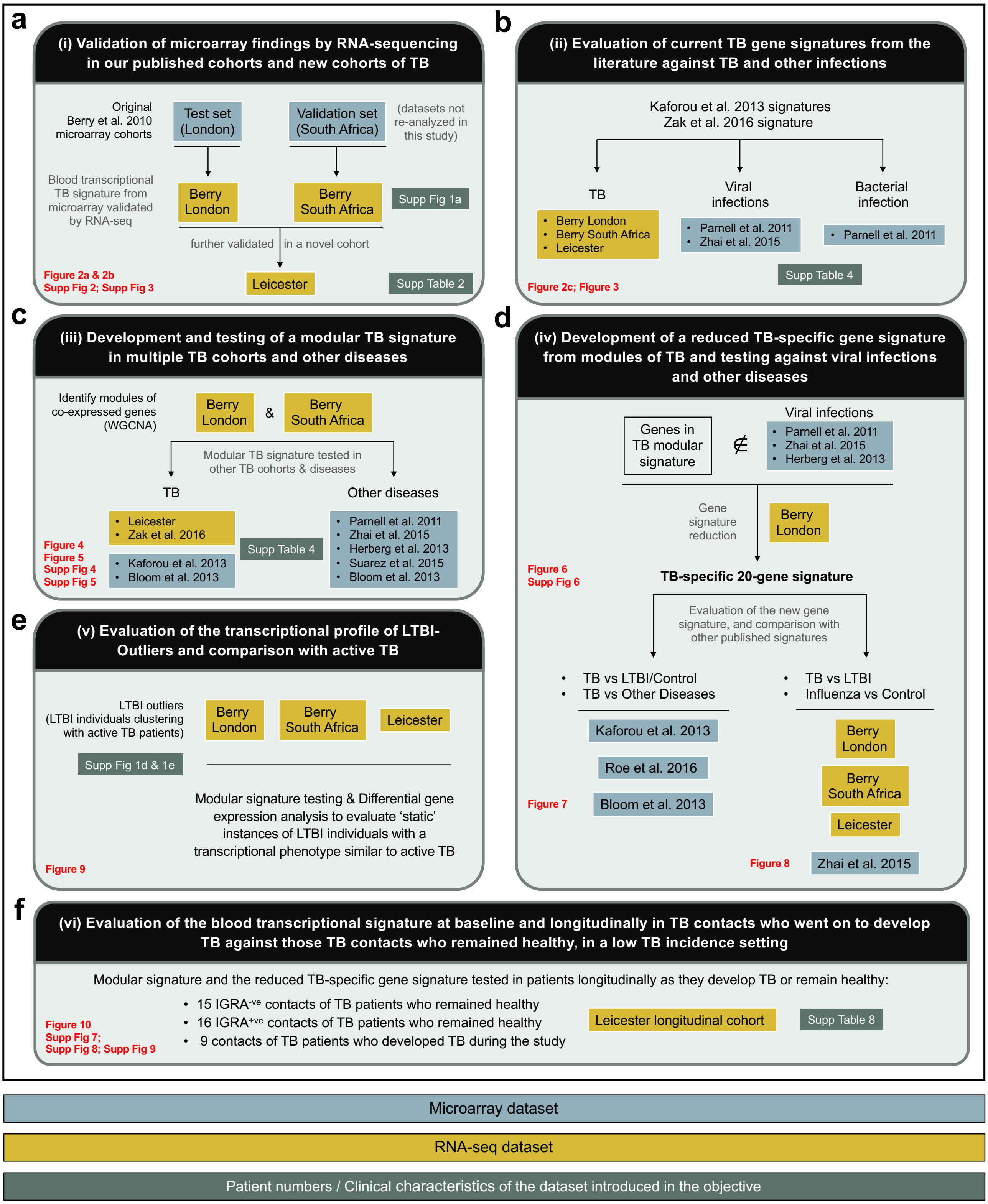

Supplementary Figure 2. Whole-blood transcriptional gene signatures in TB using RNA-seq and validation of microarray findings. a Samples from the test (Berry London) and validation (Berry South Africa) sets from Berry et al. 2010[1], originally profiled using microarray in the publication, were profiled in this present study using RNA-sequencing. **b** Heatmaps depicting unsupervised hierarchical clustering of active TB (red), LTBI (black) and control samples (purple) using the microarray 393-transcript signature of TB from Berry et al. 2010[1] in RNA-seq samples from Berry London and Berry South Africa cohorts. **c** Volcano plots depicting the 373-gene signature of TB derived independently using RNA-seq Berry London cohort. **d** Outliers identified by unsupervised hierarchical clustering in the Berry London and Berry South Africa cohorts, and **(e)** Leicester cohort.

a RNA-seq on Berry et al. 2010 samples

Group	Test set (London) (n=54)	Validation set (South Africa) (n=47)
Controls	12	-
LTBI	21	31
Active TB	21	16

b

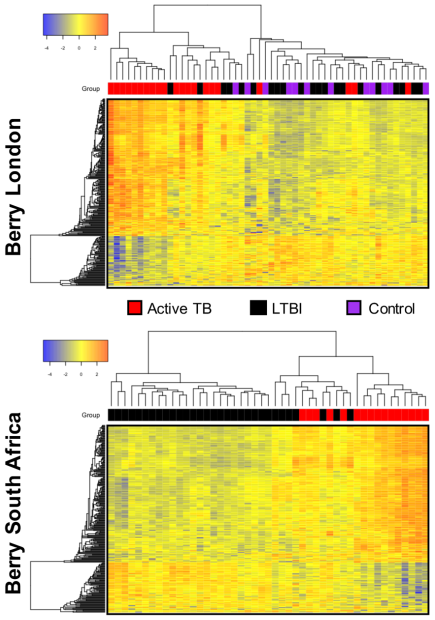

c

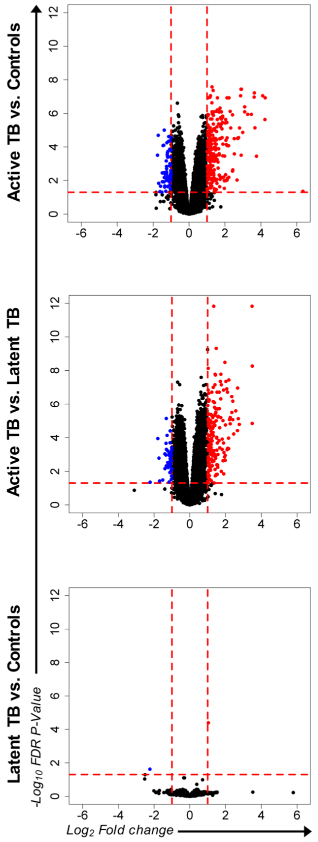

d OUTLIERS – Combined Berry London & South Africa

Group	London (n=54)	South Africa (n=47)	Combined London & South Africa (n=101)
Controls (without outliers)	11	-	11
Controls Outliers	1	-	1
LTBI (without outliers)	16	25	41
LTBI–Outliers	5	6	11
Active TB (without outliers)	17	16	33
Active TB Outliers	4	-	4

e OUTLIERS – Leicester cohort

Group	Leicester (n=152)
Controls (without outliers)	49
Controls Outliers	1
LTBI (without outliers)	44
LTBI–Outliers	5
Active TB (without outliers)	42
Active TB Outliers	11

Supplementary Figure 3. Cellular deconvolution analysis in whole-blood RNA-seq TB samples. a Stacked bar plots depicting *in silico* cellular composition of whole-blood RNA-seq samples from Berry London, **(b)** Berry South Africa and **(c)** Leicester cohorts derived using CIBERSORT. Percent fractions for 11 representative cell types for each sample are shown, with colours representing the different cell types. **d** CIBERSORT fractions for CD4, CD8, B cells, Monocytes/Macrophages and Neutrophils compared between active TB, LTBI and controls in Berry London cohort. Mean values are shown as a line in each group. p-values were calculated using a one-way ANOVA, with Tukey multiple test correction. *, p≤0.05; **, p≤0.01; ***, p≤0.001.

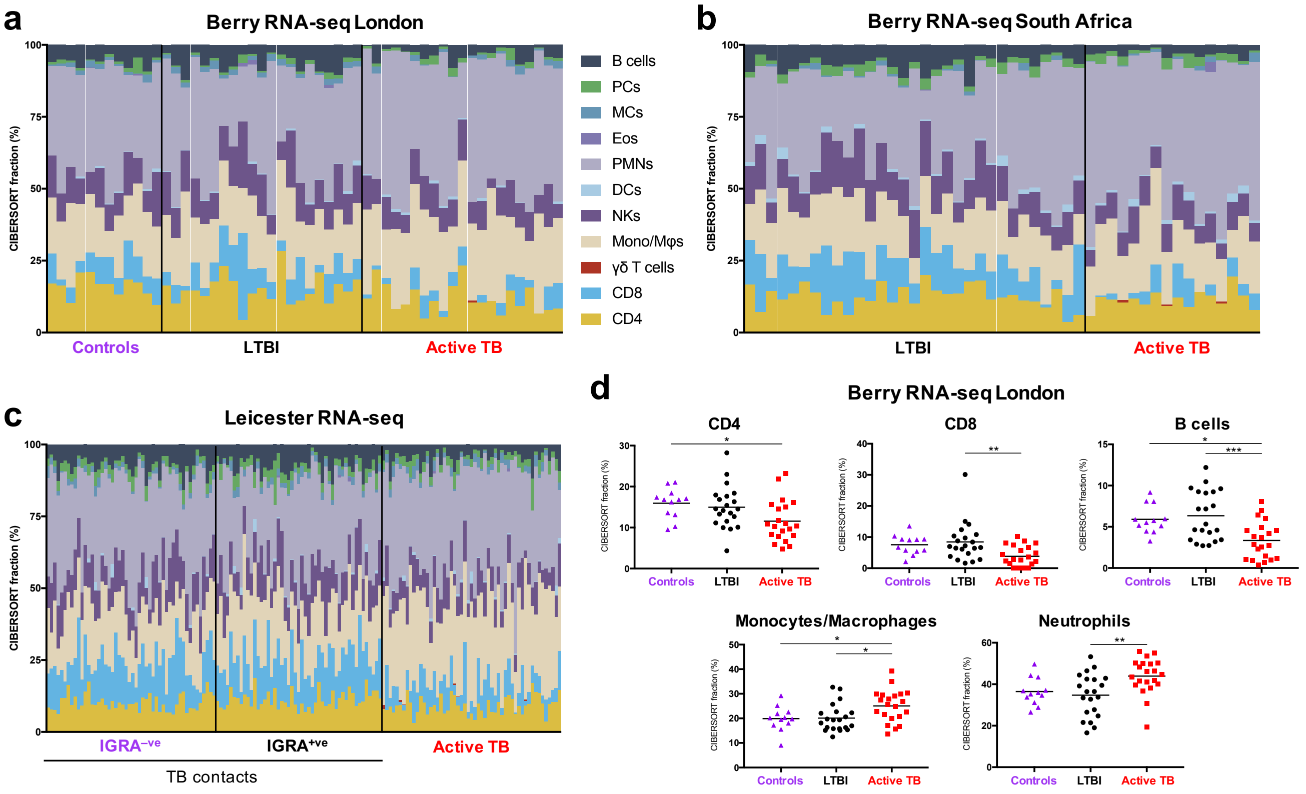

Supplementary Figure 4. Differential expression of published gene signatures in TB compared to severe influenza infection over time. a Log_2 fold changes in Berry London cohort (derived from active TB vs. controls; *y-axis*) compared to log_2 fold changes derived from severe influenza vs. controls from Parnell et al.2011[2] (*x-axis*) for genes from the 16-gene signature from Zak et al. 2016[3], and **(b)** the 27-gene signature (TB vs. LTBI) and **(c)** 44-gene signature (TB vs. other diseases (OD)) from Kaforou et al. 2013[4]. Colours represent the WGCNA module membership of the gene. Shapes represent significance associated with the fold changes (FDR p-value < 0.05) in either Berry London only (squares), respective dataset only (diamonds), both (circles) or neither (triangles).

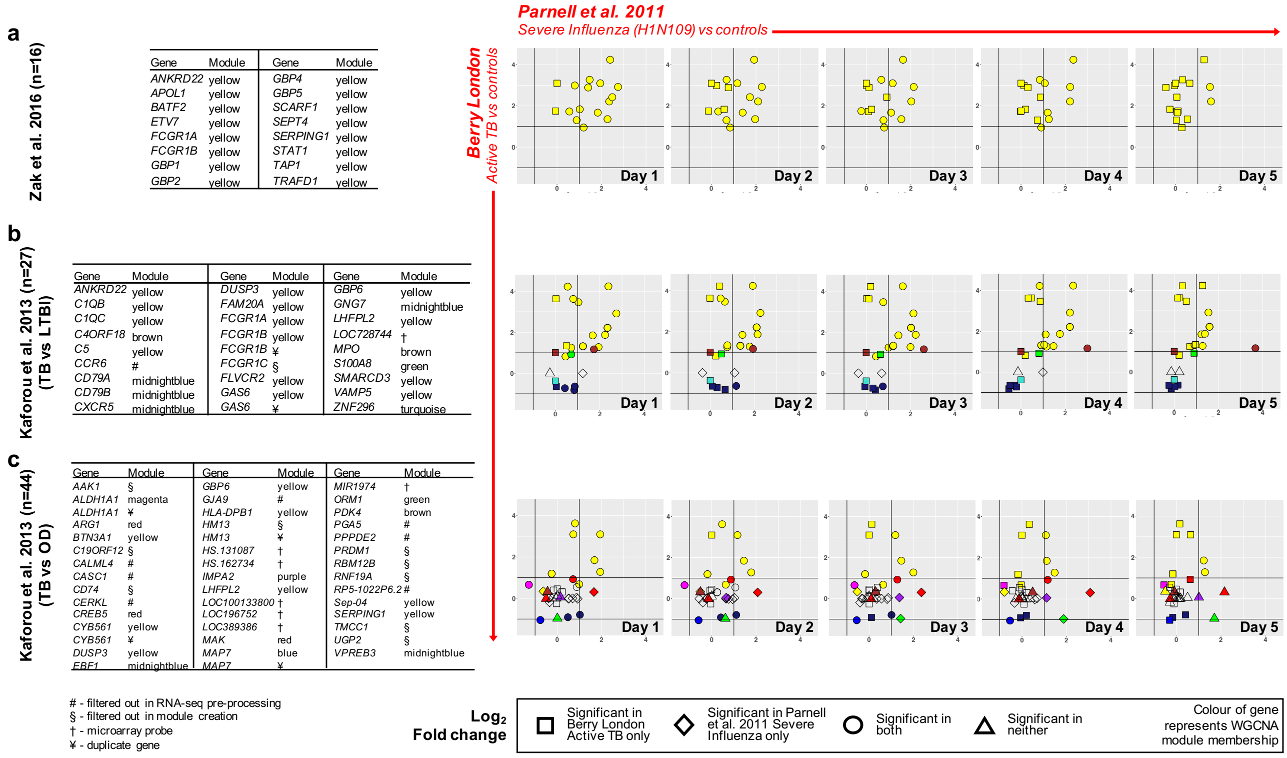

a

Gene	Module	Gene	Module
ANKRD22	yellow	*GBP4*	yellow
APOL1	yellow	*GBP5*	yellow
BATF2	yellow	*SCARF1*	yellow
ETV7	yellow	*SEPT4*	yellow
FCGR1A	yellow	*SERPING1*	yellow
FCGR1B	yellow	*STAT1*	yellow
GBP1	yellow	*TAP1*	yellow
GBP2	yellow	*TRAFD1*	yellow

b

Gene	Module	Gene	Module	Gene	Module
ANKRD22	yellow	*DUSP3*	yellow	*GBP6*	yellow
C1QB	yellow	*FAM20A*	yellow	*GNG7*	midnightblue
C1QC	yellow	*FCGR1A*	yellow	*LHFPL2*	yellow
C4ORF18	brown	*FCGR1B*	yellow	*LOC728744*	†
C5	yellow	*FCGR1B*	¥	*MPO*	brown
CCR6	#	*FCGR1C*	§	*S100A8*	green
CD79A	midnightblue	*FLVCR2*	yellow	*SMARCD3*	yellow
CD79B	midnightblue	*GAS6*	yellow	*VAMP5*	yellow
CXCR5	midnightblue	*GAS6*	¥	*ZNF296*	turquoise

c

Gene	Module	Gene	Module	Gene	Module
AAK1	§	*GBP6*	yellow	*MIR1974*	†
ALDH1A1	magenta	*GJA9*	#	*ORM1*	green
ALDH1A1	¥	*HLA-DPB1*	yellow	*PDK4*	brown
ARG1	red	*HM13*	§	*PGA5*	#
BTN3A1	yellow	*HM13*	¥	*PPPDE2*	#
C19ORF12	§	*HS.131087*	†	*PRDM1*	§
CALML4	#	*HS.162734*	†	*RBM12B*	§
CASC1	#	*IMPA2*	purple	*RNF19A*	§
CD74	§	*LHFPL2*	yellow	*RP5-1022P6.2*	#
CERKL	#	*LOC100133800*	†	*Sep-04*	yellow
CREB5	red	*LOC196752*	†	*SERPING1*	yellow
CYB561	yellow	*LOC389386*	†	*TMCC1*	§
CYB561	¥	*MAK*	red	*UGP2*	§
DUSP3	yellow	*MAP7*	blue	*VPREB3*	midnightblue
EBF1	midnightblue	*MAP7*	¥		

Supplementary Figure 5. Differential expression of published gene signatures in TB compared to influenza A infection over time. a Log_2 fold changes in Berry London cohort (derived from active TB vs. controls; *y-axis*) compared to log_2 fold changes derived from influenza A vs. controls from Zhai et al. 2015[5] (*x-axis*) for genes from the 16-gene signature from Zak et al.2016[3], and **(b)** the 27-gene signature (TB vs. LTBI) and **(c)** 44-gene signature (TB vs. other diseases (OD)) from Kaforou et al.2013[4]. Colours represent the WGCNA module membership of the gene. Shapes represent significance associated with the fold changes (FDR p-value < 0.05) in either Berry London only (squares), respective dataset only (diamonds), both (circles) or neither (triangles).

a Zak et al. 2016 (n=16)

Gene	Module	Gene	Module
ANKRD22	yellow	*GBP4*	yellow
APOL1	yellow	*GBP5*	yellow
BATF2	yellow	*SCARF1*	yellow
ETV7	yellow	*SEPT4*	yellow
FCGR1A	yellow	*SERPING1*	yellow
FCGR1B	yellow	*STAT1*	yellow
GBP1	yellow	*TAP1*	yellow
GBP2	yellow	*TRAFD1*	yellow

b Kaforou et al. 2013 (n=27) (TB vs LTBI)

Gene	Module	Gene	Module	Gene	Module
ANKRD22	yellow	*DUSP3*	yellow	*GBP6*	yellow
C1QB	yellow	*FAM20A*	yellow	*GNG7*	midnightblue
C1QC	yellow	*FCGR1A*	yellow	*LHFPL2*	yellow
C4ORF18	brown	*FCGR1B*	yellow	*LOC728744*	†
C5	yellow	*FCGR1B*	¥	*MPO*	brown
CCR6	#	*FCGR1C*	§	*S100A8*	green
CD79A	midnightblue	*FLVCR2*	yellow	*SMARCD3*	yellow
CD79B	midnightblue	*GAS6*	yellow	*VAMP5*	yellow
CXCR5	midnightblue	*GAS6*	¥	*ZNF296*	turquoise

c Kaforou et al. 2013 (n=44) (TB vs OD)

Gene	Module	Gene	Module	Gene	Module
AAK1	§	*GBP6*	yellow	*MIR1974*	†
ALDH1A1	magenta	*GJA9*	#	*ORM1*	green
ALDH1A1	¥	*HLA-DPB1*	yellow	*PDK4*	brown
ARG1	red	*HM13*	§	*PGA5*	#
BTN3A1	yellow	*HM13*	¥	*PPPDE2*	#
C19ORF12	§	*HS.131087*	†	*PRDM1*	§
CALML4	#	*HS.162734*	†	*RBM12B*	§
CASC1	#	*IMPA2*	purple	*RNF19A*	§
CD74	§	*LHFPL2*	yellow	*RP5-1022P6.2*	#
CERKL	#	*LOC100133800*	†	*Sep-04*	yellow
CREB5	red	*LOC196752*	†	*SERPING1*	yellow
CYB561	yellow	*LOC389386*	†	*TMCC1*	§
CYB561	¥	*MAK*	red	*UGP2*	§
DUSP3	yellow	*MAP7*	blue	*VPREB3*	midnightblue
EBF1	midnightblue	*MAP7*	¥		

\# - filtered out in RNA-seq pre-processing
§ - filtered out in module creation
† - microarray probe
¥ - duplicate gene

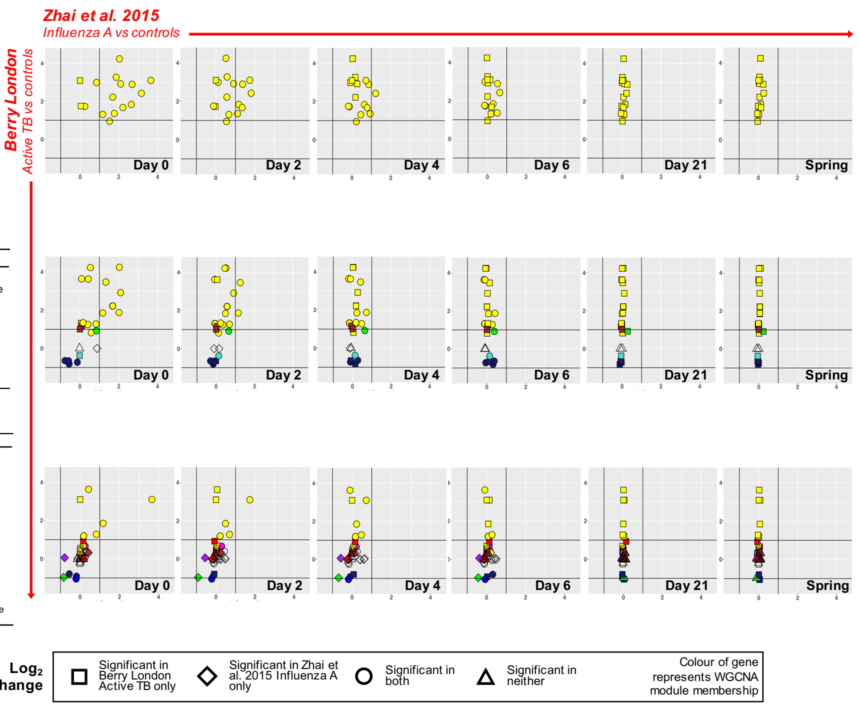

Supplementary Figure 6. Development of a reduced TB-specific gene signature. a Representative TB modular profile depicting modules that were perturbed in TB. **b** Venn diagram depicting genes significantly differentially expressed in TB only (active TB vs. controls in Berry London cohort) and not in influenza (influenza vs. controls in Parnell et al. 2011[2], Zhai et al. 2015[5] and Herberg et al. 2013[6]). **c** The 61-gene signature identified as predictive of active TB compared to LTBI using the Boruta algorithm. **d** The set of 20 genes with the highest GINI score (using random forest) in the Boruta 61-gene signature. Module membership of the 20-gene signature is also shown.

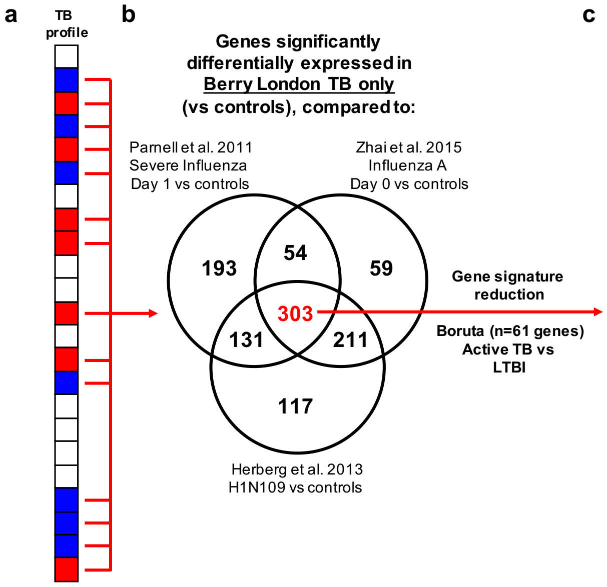

c

Gene	Module
ARHGEF9	blue
ATP8B2	blue
BCL11B	blue
BDH1	blue
C1QTNF3	blue
CATSPER2	blue
DCHS1	blue
GGT7	blue
KCNC4	blue
KLHL25	blue
OLFM2	blue
PAIP2B	blue
SLC2A11	blue
SMYD5	blue
TMEM25	blue
TRMT11	blue
VEGFB	blue
ZNF391	blue
CAPZA1	brown
GALNT3	brown
MED12L	brown
FAM84B	cyan
ZNF549	cyan
ZNF251	darkred
SELP	greenyellow
SPHK1	greenyellow
EFCAB2	magenta
ICAM1	magenta
BACH2	midnightblue
STARD9	midnightblue
ZNF525	salmon

Gene	Module
TTC22	tan
CCDC120	turquoise
COL4A4	turquoise
IGSF8	turquoise
KLC2	turquoise
NEIL1	turquoise
PGBD1	turquoise
SAMD10	turquoise
SDC2	turquoise
SLC16A11	turquoise
SLC25A45	turquoise
TCTN2	turquoise
TRAF4	turquoise
TREX2	turquoise
TRIM47	turquoise
USP54	turquoise
ZAP70	turquoise
APOL4	yellow
ARNTL2	yellow
ARSB	yellow
ATF3	yellow
CTSB	yellow
FANCA	yellow
FZD5	yellow
KIAA1522	yellow
LIMK1	yellow
OLFML2B	yellow
SCARF1	yellow
SRC	yellow
TRPM4	yellow

Top 20 genes with highest GINI score (Random Forest)

d

Gene	Module
ARHGEF9	blue
BDH1	blue
KCNC4	blue
PAIP2B	blue
SMYD5	blue
TMEM25	blue
VEGFB	blue
SPHK1	greenyellow
ICAM1	magenta
BACH2	midnightblue
CCDC120	turquoise
COL4A4	turquoise
TRAF4	turquoise
TRIM47	turquoise
USP54	turquoise
APOL4	yellow
ARNTL2	yellow
CTSB	yellow
LIMK1	yellow
SCARF1	yellow

Supplementary Figure 7. Modular transcriptional profiles of TB contacts followed over time. Longitudinal modular profiles of $IGRA^{-ve}$ TB contacts who remained healthy (n=15), $IGRA^{+ve}$ TB contacts who remained healthy (n=16), and TB contacts who developed TB during the study (n=9). Enrichment scores derived using ssGSEA compared to the average enrichment scores of $IGRA^{-ve}$ controls are depicted, with red and blue indicating modules over- or under-expressed compared to controls. Colour intensity and size represent the degree of enrichment. The x-axis depicts the time in months of recruitment for the study from Baseline for each patient. For the contacts who developed TB during the study, the time point when the contact was diagnosed with active TB in the clinic is represented in red letters. Representative modular TB profiles depicting modules that were perturbed in TB are shown for visual comparison on either side of each modular figure.

Supplementary Figure 7.

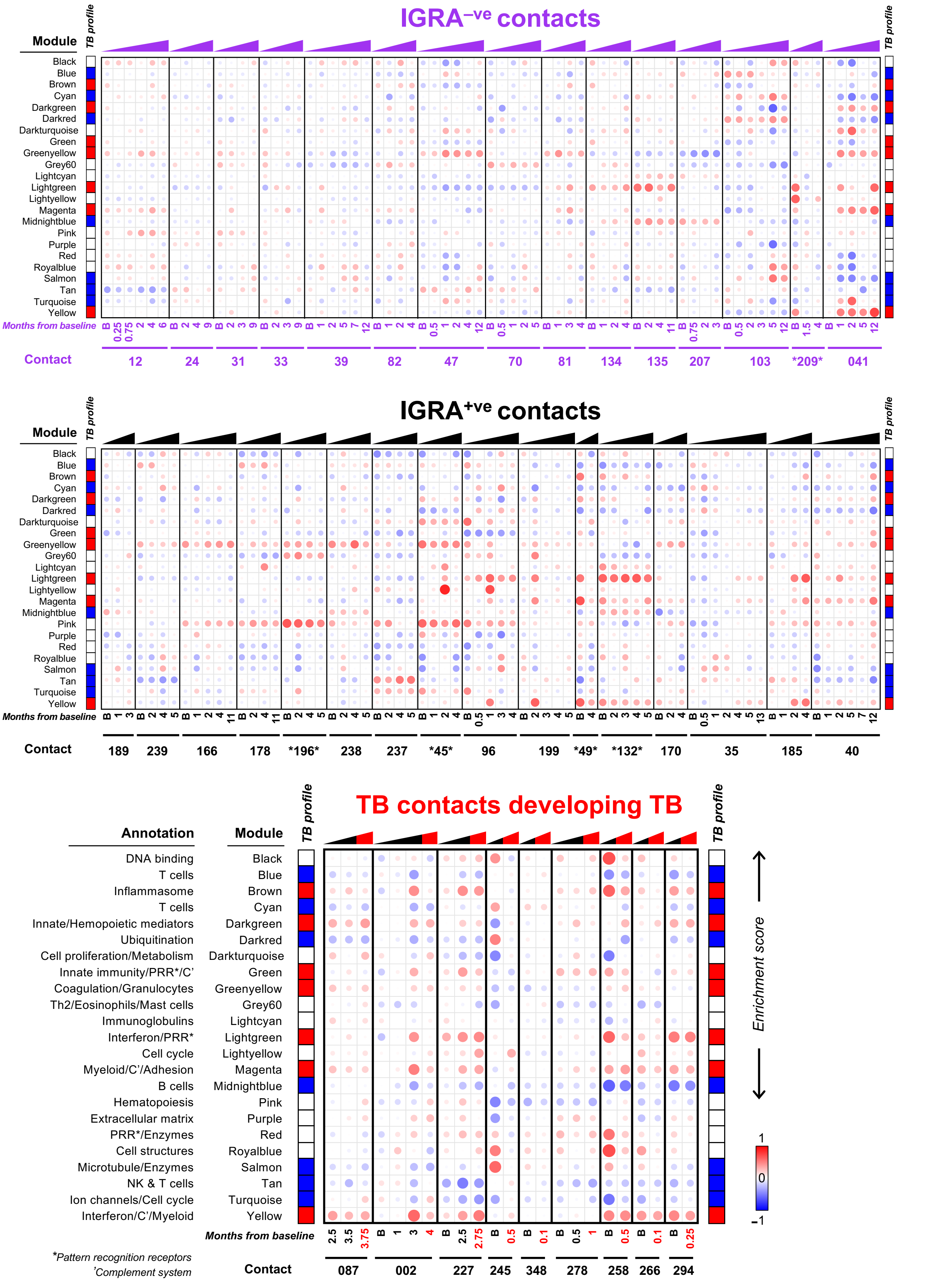

Supplementary Figure 8. Baseline visit scores in TB contacts followed over time. Scatter plots depicting modified Disease Risk scores at Baseline visit in IGRA^{-ve} and IGRA^{+ve} TB contacts who remained healthy, and in TB contacts who developed TB during the study (n=9) using the 20-gene TB-specific signature. The dispersion is expressed as median with 95% CI. Outliers are indicated as empty circles (IGRA^{-ve}) or squares (IGRA^{+ve}) with a dot inside.

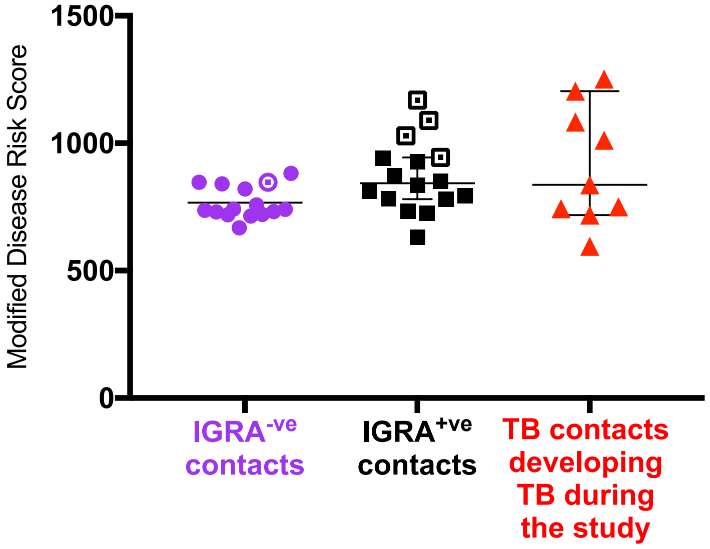

Supplementary Figure 9. Blood transcriptional profile of TB contacts followed over time using the Zak et al. 2016 signature. a Bar plots depicting the modified Disease Risk Scores using the Zak et al. 2016[3] 16-gene signature, in TB contacts who remained IGRA^{-ve} and did not develop TB (n=15), TB contacts who remained IGRA^{+ve} and did not develop TB (n=16), and TB contacts who developed TB during the study (n=9). For TB contacts who developed TB during the study, the time point when the contact was diagnosed with active TB in the clinic is represented by a red bar. Baseline in the barplot is set at 1824.35, average of all Baseline time-point modified Disease Risk Scores from all IGRA^{-ve} contacts (n=15).

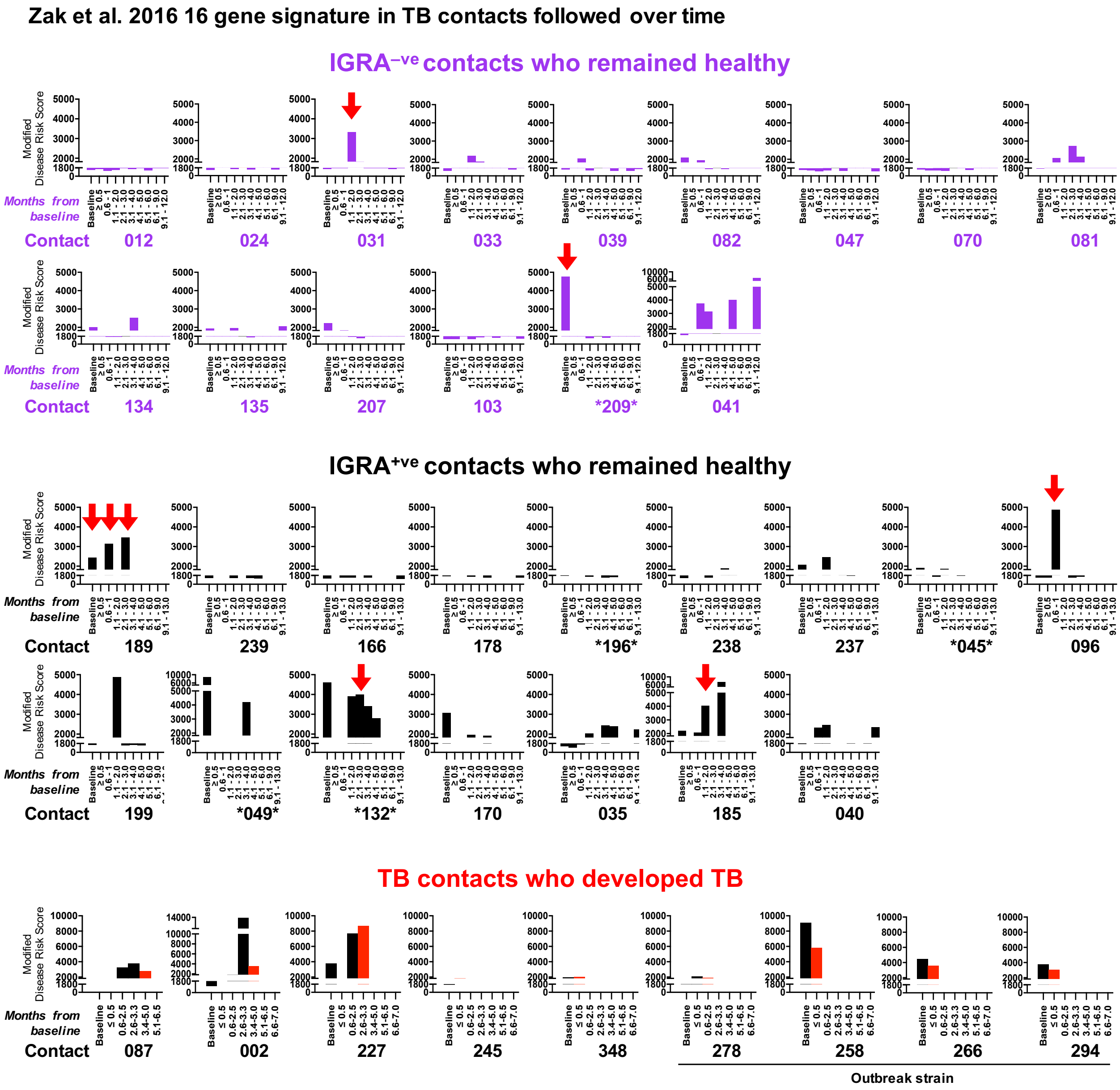

Supplementary Tables

Supplementary Table 1. Clinical characteristics of IGRA^{-ve} TB contacts, IGRA^{+ve} TB contacts and Active TB patients recruited in Leicester.

DEMOGRAPHICS		ACTIVE TB	TB CONTACTS	
			IGRA Positive	IGRA Negative
		(*n* = 54*)	(*n* = 50#)	(*n* = 50)
GENDER	Male (%)	35 (65)	29 (58)	30 (60)
	Female (%)	19 (35)	21 (42)	20 (40)
AGE	years (mean ±SD)	40 (± 15)	39 (± 15)	35 (± 16)
ETHNICITY	Indian Subcontinent (%)	34 (63)	39 (78)	45 (90)
	African (%)	11 (20)	7 (14)	4 (8)
	White Caucasian (%)	9 (17)	4 (8)	1 (2)
ORIGIN	UK born (%)	8 (15)	4 (8)	10 (20)
	Foreign born (%)	46 (75)	46 (92)	40 (80)
DISEASE TYPE$	Smear positive pulmonary (%)	20 (37)	16 (32)	10 (20)
	Smear negative pulmonary (%)	24 (44)	21 (42)	0
	Non-pulmonary (%)	10 (19)	13 (26)	40 (80)
DURATION OF SYMPTOMS	months (mean ±SD)	2.13 (± 1.62)		
CONTACT TYPE	Household, partner (%)		8 (16)	8 (16)
	Household, other (%)		36 (72)	39 (78)
	Non-household (%)		6 (12)	3 (6)

*One patient removed as part of quality control

#One patient not sequenced

$ For TB contacts disease type refers to the phenotype of TB in the index case to which the contact was exposed

SD – Standard deviation

Supplementary Table 2. TB gene signatures from Zak et al. 2016 and Kaforou et al. 2013.

Zak et al. 2016 (n=16)		Kaforou et al. 2013			
		TB vs. LTBI (n=27)		TB vs. OD (n=44)	
Genes	IFN inducible (n=16)	Genes	IFN inducible (n=18)	Genes	IFN inducible (n=22)
ANKRD22	*	ANKRD22	*	AAK1	*
APOL1	*	C1QB	*	ALDH1A1	*
BATF2	*	C1QC	*	ALDH1A1	*
ETV7	*	C4ORF18		ARG1	
FCGR1A	*	C5	*	BTN3A1	*
FCGR1B	*	CCR6		C19ORF12	*
GBP1	*	CD79A		CALML4	*
GBP2	*	CD79B		CASC1	
GBP4	*	CXCR5		CD74	*
GBP5	*	DUSP3	*	CERKL	
SCARF1	*	FAM20A	*	CREB5	*
SEPT4	*	FCGR1A	*	CYB561	
SERPING1	*	FCGR1B	*	CYB561	
STAT1	*	FCGR1B	*	DUSP3	*
TAP1	*	FCGR1C	*	EBF1	
TRAFD1	*	FLVCR2	*	GBP6	*
		GAS6	*	GJA9	
		GAS6	*	HLA-DPB1	*
		GBP6	*	HM13	
		GNG7		HM13	
		LHFPL2		HS.131087	
		LOC728744		HS.162734	
		MPO		IMPA2	*
		S100A8	*	LHFPL2	
		SMARCD3	*	LOC100133800	
		VAMP5	*	LOC196752	
		ZNF296	*	LOC389386	
				MAK	*
				MAP7	*
				MAP7	*
				MIR1974	
				ORM1	
				PDK4	*
				PGA5	
				PPPDE2	
				PRDM1	*
				RBM12B	
				RNF19A	*
				RP5-1022P6.2	
				SEPT4	*
				SERPING1	*
				TMCC1	*
				UGP2	*
				VPREB3	

*IFN inducible genes, as identified by the Interferome database v2.01

Supplementary Table 3. Numbers of subjects in the external datasets downloaded from GEO.

Kaforou et al. 2013 (GSE37250)

Group	n
Active TB (HIV-/+)	195
Latent TB (HIV-/+)	167

Zak et al. 2016 (GSE79362)

Group		n
Days before diagnosis	1 to 180	19
	181 to 360	29
	361 to 540	17
	541 to 720	11
	>720	4
Non-progressors		243

Parnell et al. 2011 (GSE20346)

Group		n
Severe Influenza	Day 1	4
	Day 2	4
	Day 3	4
	Day 4	4
	Day 5	3
Bacterial Pneumonia	Day 1	6
	Day 2	6
	Day 3	6
	Day 4	4
	Day 5	4
Controls		18

Zhai et al. 2015 (GSE68310)

Group		n
Influenza A	Day 0	42
	Day 2	45
	Day 4	41
	Day 6	42
	Day 21	45
	Spring	42
Controls		44

Herberg et al. 2013 (GSE42026)

Group	n
H1N109	19
RSV	22
Bacteria	18
Controls	33

Suarez et al. 2015 (GSE60244)

Group	n
Virus	71
Bacteria	22
Coinfection	25
Controls	40

Bloom et al. 2013 (GSE42834)

Group	n
Active TB	35
Sarcoidosis	61
Bacterial Pneumonia	14
Lung cancer	16
Controls	113

Supplementary Table 4. Annotation of 23 modules of co-expressed genes derived using WGCNA from combined Berry London & South Africa cohorts.

Module	Number of genes	Annotation
Black	203	DNA-binding proteins, Ubiquitination
Blue	902	T-lymphocytes, Antigens
Brown	428	Inflammasome pathway, IL-10 signaling, TLR signaling, Monocytes
Cyan	121	T-lymphocytes, Molecular conformation
Darkgreen	27	Lymphotoxin β Receptor Signaling, Endosomes
Darkred	38	Ubiquitination, Mutagenesis
Darkturquoise	27	Cell proliferation & Metabolism
Green	267	Chemokine signaling, Complement system, Neutrophils, Monocytes, Interleukins, Pattern recognition receptors
Greenyellow	159	Coagulation system, Granulocyte Adhesion and Diapedesis
Grey60	94	Th2 pathway, Eosinophils, Mast cells
Lightcyan	98	Immunoglobulins
Lightgreen	86	Interferon signaling, Interferon Type-I, Pattern recognition receptors
Lightyellow	59	Cell cycle, cytoskeleton proteins
Magenta	165	Glycoproteins/Antigens, Macrophages/Monocytes, Complement, Cell adhesion
Midnightblue	108	B cell receptor signaling
Pink	167	Hematopoiesis, B cell development, Ubiquitination, Hemoglobins
Purple	165	Extracellular matrix proteins, cell differentiation
Red	236	Pattern recognition receptors, Enzymes and Coenzymes
Royalblue	38	Cellular structures, Cell surface receptors, Membrane proteins
Salmon	136	Microtubule proteins, Regulatory elements, Enzymes and Coenzymes
Tan	157	Antigens and Receptors, Lymphocytes, Th1 and Th2 activation pathway, NK cell signaling
Turquoise	959	Ion channels, cell cycle proteins
Yellow	360	Interferon signaling, Complement system, MHC & Antigen presentation, Dendritic cells/Macrophages

Supplementary Table 5. Clinical characteristics of the TB contacts identified with TB during prospective observation in the Leicester Cohort.
The table summarises longitudinal clinical observations recorded for each subject until TB diagnosis (marked in red on longitudinal time course)

Study Number	002	227	087	245	348	278	258	266	294
**Clinical and Demographic Characteristics**									
Gender	Female	Female	Male	Male	Female	Male	Male	Male	Male
Ethnicity	Indian Subcontinent	African	Indian Subcontinent	European	White Caucasian	White Caucasian	White Caucasian	White Caucasian	White Caucasian
Birth Origin	Foreign Born	Foreign Born	Foreign Born	Foreign Born	UK Born	UK Born	UK Born	UK Born	UK Born
Index Disease Type	Smear positive Pulmonary TB	Smear positive Pulmonary TB	Smear negative Pulmonary TB	Smear negative Pulmonary TB	Smear negative Pulmonary TB	Smear negative Pulmonary TB	Smear negative Pulmonary TB	Smear negative Pulmonary TB	Smear negative Pulmonary TB
Type of Contact	Partner	Household	Household	Household	Household	Partner	Partner	Household	Household
Contact Disease Type	Non-Pulmonary TB	Pulmonary TB	Pulmonary TB	Pulmonary TB	Pulmonary TB	Pulmonary TB	Pulmonary TB	Pulmonary TB	Pulmonary TB
Category of TB Infection	True progressor	True progressor	Subclinical active TB	Subclinical active TB	Active TB	Active TB	Active TB	Active TB	Active TB
IGRA Result	Positive	Positive	Positiive	Positive	Positive	Positive	Positive	Positive	Negative
Culture status<br>Culture positive sample TTPC (days)	Positive<br>Pleural fluid<br>21	Positive<br>BAL<br>14	Positive<br>Sputum<br>6	Positive<br>Sputum<br>21	Positive<br>Sputum<br>20	Positive<br>Sputum<br>20	Positive<br>Sputum<br>8	Positive<br>Sputum<br>12	Positive<br>Sputum<br>21
Xpert MTB/RIF assay	Negative	Positive	Positive	Positive	Positive	Negative	Positive	Positive	Positive
**Longitudinal time course**									
Time course (Months after baseline)	B 1 3 4	B 2.5 2.75	B 2.5 3.5 3.75	B 0.5	B 0.1	B 0.5 1	B 0.5	B 0.1	B 0.25
Reported Symptoms	X X ۞۞	X ۞	X X ۞	X ۞۞۞	۞	۞	۞	۞۞	۞۞
CXR Appearance	N ‡	N ‡	‡	‡	‡	‡	‡	‡	‡
Other Clinical Information	Nil	Nil	Also sputum culture positive for *S.Pneumoniae*	Received antibiotics for suspected bacterial pneumonia	Nil	Nil	Nil	Nil	Nil

* TTPC – Time to positive culture
^ BAL - Bronchoalveolar lavage
X – No reported symptoms at clinical assessment
۞ – Reported symptom compatible with active TB (including cough, fever and weight loss). Each symbol refers to a single symptom type.
N – Normal chest X-ray (reported by Consultant thoracic radiologist)
‡ - Abnormal chest X-ray with TB associated features

References

1 Berry, M. P. *et al*. An interferon-inducible neutrophil-driven blood transcriptional signature in human tuberculosis. *Nature* **466**, 973-977, doi:10.1038/nature09247 (2010).

2 Parnell, G. *et al*. Aberrant cell cycle and apoptotic changes characterise severe influenza A infection–a meta-analysis of genomic signatures in circulating leukocytes. *PLOS One* **6**, e17186 (2011).

3 Zak, D. E. *et al*. A blood RNA signature for tuberculosis disease risk: a prospective cohort study. *The Lancet* **387**, 2312-2322 (2016).

4 Kaforou, M. *et al*. Detection of tuberculosis in HIV-infected and-uninfected African adults using whole blood RNA expression signatures: a case-control study. *PLOS Med* **10**, e1001538 (2013).

5 Zhai, Y. *et al*. Host transcriptional response to influenza and other acute respiratory viral infections–a prospective cohort study. *PLOS Pathog* **11**, e1004869 (2015).

6 Herberg, J. A. *et al*. Transcriptomic profiling in childhood H1N1/09 influenza reveals reduced expression of protein synthesis genes. *J Infect Dis* **208**, 1664-1668 (2013).
