# Supplementary material for: A modular transcriptional signature identifies phenotypic heterogeneity of human tuberculosis infection
Source: Nat Commun. 2018 Jun 19;9:2308. doi: 10.1038/s41467-018-04579-w (PMC6008327; doi:10.1038/s41467-018-04579-w)
Supplement: Supplementary file 2 — Description of Additional Supplementary Files [file 41467_2018_4579_MOESM2_ESM.pdf]

Description of Additional Supplementary Files

File Name: **Supplementary Data 1**
Description: The whole-blood 373-gene transcriptional signature of TB derived from RNA-seq Berry London cohort.

File Name: **Supplementary Data 2**
Description: List of co-expressed genes in the 23 modules derived using WGCNA from combined Berry London & South Africa cohorts.

File Name: **Supplementary Data 3**
Description: List of the 70 genes consistently upregulated in active TB (without outliers) and LTBI outliers from Berry Combined and Leicester datasets, compared to respective controls.
